# Iodide of Sodium in Constitutional Syphilis

**Published:** 1853-03

**Authors:** 


*Iodide of Sodium in Constitutional Syphilis.*—Dr. Gamberini, of Bologna, has written a paper on the use of iodide of sodium in the treatment of constitutional syphilis, which he concludes with the following resumè:

1. Soda being a very common ingredient in our organism, the iodide of its base appears best suited to the human system.

2. The taste of the iodide of sodium is much less disagreeable than that of iodide of potassium.

3. It is less likely to occasion iodism.

4. It is better borne than the potassium salt; and in consequence of this, its dose can be almost daily increased, and it thus becomes a more efficient remedy.

5. It has sometimes succeeded, when the iodide of potassium had failed.

6. We may commence by giving daily, in three equal doses, a scruple of the salt dissolved in three ounces of distilled water, increasing the strength of the solution every two or three days by six grains. More than two drachms a day are thus sometimes taken without inconvenience.

7. The iodide of sodium is admirably adapted to the cases in which the corresponding salt of potassium is indicated.

8. The iodide of sodium is the best substitute for mercury.—*Dublin Quart. Journal,* Nov. 1852.

